# Biomarkers to Personalize the Treatment of Rheumatoid Arthritis: Focus on Autoantibodies and Pharmacogenetics

**DOI:** 10.3390/biom10121672

**Published:** 2020-12-14

**Authors:** Valeria Conti, Graziamaria Corbi, Maria Costantino, Emanuela De Bellis, Valentina Manzo, Carmine Sellitto, Berenice Stefanelli, Francesca Colucci, Amelia Filippelli

**Affiliations:** 1 Department of Medicine, Surgery and Dentistry “Scuola Medica Salernitana”, University of Salerno, 84081 Baronissi, Italy; mcostantino@unisa.it (M.C.); e.debellis93@gmail.com (E.D.B.); vmanzo@unisa.it (V.M.); csellitto@unisa.it (C.S.); berenicestefanelli@gmail.com (B.S.); f.colucci16@studenti.unisa.it (F.C.); afilippelli@unisa.it (A.F.); 2 Department of Medicine and Health Sciences, University of Molise, 86100 Campobasso, Italy; graziamaria.corbi@unimol.it; 3 Association Non-Profit F.I.R.S.Thermae (Interdisciplinary Training, Researches and Spa Sciences) in Italian National Register of Research of MIUR, Naples, 80070 Bacoli, Italy

**Keywords:** autoimmune disorder, pharmacogenetics, methotrexate, disease-modifying antirheumatic drugs, autoantibodies

## Abstract

Rheumatoid arthritis (RA) is a chronic inflammatory disease that is very complex and heterogeneous. If not adequately treated, RA patients are likely to manifest excess of morbidity and disability with an important impact on the quality of life. Pharmacological treatment is based on the administration of the disease-modifying antirheumatic drugs (DMARDs), subdivided into conventional synthetic (csDMARDs), targeted synthetic (tsDMARDs), and biological (bDMARDs). bDMARDs are now frequently administered in patients, both as alternative treatment and together with csDMARDs. Unfortunately, there is a therapeutic response variability both to old and new drugs. Therefore, to identify pre-therapeutic and on-treatment predictors of response is a priority. This review aims to summarize recent advances in understanding the causes of the variability in treatment response in RA, with particular attention to predictive potential of autoantibodies and DMARD pharmacogenetics. In recent years, several biomarkers have been proposed to personalize the therapy. Unfortunately, a magic bullet does not exist, as many factors concur to disease susceptibility and treatment outcomes, acting around the patient’s congenital background. Models integrating demographic, clinical, biochemical, and genetic data are needed to enhance the predictive capacity of specific factors singularly considered to optimize RA treatment in light of multidisciplinary patient management.

## 1. Introduction

Rheumatoid arthritis (RA) is a chronic inflammatory, autoimmune disorder affecting nearly 1% of the general population [1]. Women suffer from RA three times more often than men. Indeed, the prevalence of immune-mediated inflammatory diseases is lower in male subjects, and male RA patients appear to react better to specific treatments, such as anti-cytokines and antiproliferative drugs [2,3]. Possible reasons could be mainly related to a greater immune response in females than in males. Moreover, lymphocytes/monocytes from female subjects show higher immune/inflammatory reactivity, suggesting involvement of sex hormones in the regulation of the immune/inflammatory response in immune-mediated diseases [4,5]. In fact, sex hormones play an important role in regulating the immune/inflammatory response in RA [6].

RA initially affects small joints, progresses to larger joints and internal organs, ultimately causing an excess of morbidity and disability with an important impact on the quality of life (QoL). 

The therapy is based on a treat-to-target approach, which requires tight monitoring of the disease activity in order to implement a prompt correction of the therapy regimen. The ideal therapeutic target coincides with the clinical remission or, at the very least, the low activity disease (LDA) [1]. 

According to the updated recommendations for the management of RA of the European League Against Rheumatism (EULAR) [7], pharmacological therapy, based on the administration of disease-modifying antirheumatic drugs (DMARDs), should be started as soon as the diagnosis of RA is made. DMARDs may slow down or even arrest the progression of joint destruction, seeking to get the treatment target, mainly assessed by the Disease Activity Score based on 28 joint counts (DAS28) [7].

DMARDs are classified in conventional synthetic (csDMARDs), targeted synthetic (tsDMARDs), and biological (bDMARDs). All the DMARDs are indicated for both non-rheumatic and rheumatic diseases thanks to their various activities, mainly immunomodulatory and anti-inflammatory ones. 

Among csDMARDs, methotrexate (MTX) represents the mainstay of RA treatment.

MTX is an antimetabolite that interferes with the folate pathway binding to dihydrofolate reductase (DHFR) and thus inhibiting the transformation of dihydrofolic acid (FH_2_) into tetrahydrofolic acid (FH_4_), which is an important cofactor for the synthesis of nucleic acids and amino acids [8,9].

Under the last recommendation of the European Medicine Agency (EMA), MTX must be administered only once a week, starting with a low dose and then performing a titration as needed [10]. Serious side effects, including liver problems, bone marrow depression, and even death, occur when MTX is taken more often [10] The maximum weekly dose varies depending on the patient’s ethnic group, considering variables such as body weight and genetic factors [1].

Besides MTX, other csDMARDs suitable for RA are leflunomide and sulfasalazine. These are used as part of the first therapeutic strategy in patients with a contraindication or who manifest an early intolerance to MTX [1].

Today, besides the conventional options, clinicians have other therapeutic options, represented by ts- and bDMARDs. Tofacitinib, baricitinib, filgotinib, and upadacitinib are tsDMARDs that work by inhibiting the activity of one or more enzymes belonging to the Janus kinase family. The EULAR has included tsDMARDs among the drugs to administer in second- and later-line treatment with or without MTX. They are used in patients with moderate to severe RA who are non-responders or intolerant to csDMARDs [1].

Biological DMARDs include infliximab, adalimumab, etanercept, golimumab, and certolizumab pegol, which are all anti-tumor necrosis factor α (TNFα), and abatacept, rituximab, tocilizumab, and sarilumab. According to the EULAR recommendations [1], these drugs must be started in second- and third-line therapy, with or without MTX.

It is useful to combine pharmacological and non-pharmacological treatments with the promotion of a healthy and active lifestyle to reach the treatment target. However, despite huge advances in clinical management, diagnostic tool, and pharmacological therapy, not all patients reach an adequate response.

A variable response occurs, in fact, both to drugs and nonpharmacological therapy; therefore, there is a need to deeply investigate the factors involved in such variability observed in daily clinical practice [1].

This review aims to summarize recent advances in understanding the causes of the variability in treatment response observed in daily clinical practice in patients with RA, with particular attention to more recent data on autoantibodies and DMARD pharmacogenetics.

## 2. Role of Autoantibodies in Predicting the Response to RA Treatment

In RA, many antibodies, such as anti-citrullinated protein antibodies (ACPAs), rheumatoid factor (RF), and anti-carbamylated protein (anti-CarP), with diagnostic and prognostic role have been identified. All of these are principally present in RA patients’ serum and synovial fluid (SF), but ACPAs and RF are also found in lung, sputum, periodontium, intestine, and cervical–vaginal mucosa [11]. For this reason, RF and ACPAs are routinely used, mainly as biomarkers for diagnostic purpose [12]. Indeed, the European League Against Rheumatism/American College of Rheumatology (EULAR/ACR) has also introduced RF and ACPAs in the diagnostic criteria of RA, and it suggests their use in guiding the choice of treatments to prevent the development of symptomatic RA [13]. The ACPAs are the most relevant diagnostic and prognostic biomarkers for RA [14] because their determination permits to classify RA patients into seropositive versus seronegative, as well as RF measurement. The ACPAs include a group of autoantibodies directed against peptides and proteins that are present in 70–90% of RA patients and have high disease-specificity (90–95%) [15]. In fact, unlike the RF, they are rarely found in other diseases or healthy subjects. In a meta-analysis by Nishimura et al., ACPAs showed a sensitivity similar to RF (≈67% vs. ≈69%), but a higher specificity (≈95% vs. ≈85%) [16]. These autoantibodies are predictive for RA development in patients without a previous diagnosis, and for more severe disease [17]. The autoimmune response in RA is presumably initiated by citrullination of self-peptides, leading to alterations of their properties. ACPAs are locally produced in RA joints, where proteins are citrullinated during the inflammatory process [18]. Citrullination [19] leads to the activation of complex immune responses and specific ACPA generation, found in approximately 75% of RA patients [20]. The risk of developing RA also depends on hereditary factors [21], and over 100 genetic loci are involved in an increased risk of RA [22]. Interestingly, twin studies have found no difference in heritability in subsets of ACPA-positive and ACPA-negative RA [23]. Instead, an analysis of large Swedish population registers showed that inheritability accounted for around 50% of ACPA-positive RA, but only 20% of ACPA-negative RA [24]. In several studies, HLA-DRB1 alleles [25], including DRB1*0401, *0404/*0408, *0405, *0101, *1001, and *1402, presenting a similar sequence at amino acid positions 67–74 in the third hypervariable region of the DR molecule (called the “shared epitope”) were associated with RA. Similarly, susceptibility to RA is associated with certain DQB1/DQA1 combinations (DQB1*03 and *04 combined with DQA1*03 (referred to as DQ3), and DQB1*0501 combined with DQA1*0101), while RA protection is mediated by other DRB1 alleles (*0103, *0402, *1102, *1103, *1301, *1302, and *1304) [26]. In North American white people, the DRB1*0401 and *0404 alleles are the most prevalent, and these genes are associated with the highest risk of severe disease. In a recent study, Wysocki et al. underlined that HLA-DRB1 genotyping is neither used in clinical practice, nor included in the current RA classification criteria [20]. Nonetheless, some HLA-DRB1 variants seem to predict the unfavorable course of the disease, including a higher risk of radiographic damage progression, higher incidence of interstitial lung disease, or lymphoproliferative diseases. The authors also suggested the possible identification of high-risk patients bearing the HLA-DRB1 risk allele, with a consequent important role of this marker in the personalization of the therapy [20]. Analyzing data from patients with early RA treated according to different strategies, by considering DQB1 typing and DRB1 typing and subtyping, Lard et al. showed that the association between the HLA class II antigens and joint destruction is affected by early and aggressive therapy with DMARDs. This interaction was independent of other prognostic factors, such as RF and baseline disease activity. Then, the authors suggested that an early and aggressive treatment with DMARDs, such as MTX, sulfasalazine, and cloroquine, can regulate the immune process, maybe by preventing a secondary release of autoantigens, by modulating the autoantigen presentation by APCs to the CD4+ T cells, and/or by inhibiting the response of T cells [27]. RFs, first identified autoantibodies in RA, are also present in infectious diseases, wherein they facilitate the removal of pathogens, and in cancer [28,29]. RFs and ACPAs show a broad spectrum of isotypes (immunoglobulin (Ig)M, IgG, IgA) [30]. The RF is an autoantibody that recognizes domains CH2 and CH3 of the Fc portion of human immunoglobulin G (IgG). According to different studies, RF testing in RA patients has a sensitivity that varies from 60 to 90% and specificity between 48 and 92% [31]. The limited specificity of RF is due to its presence also in healthy controls and patients with other autoimmune and non-autoimmune diseases [32]; consequently, RF alone is insufficient for diagnosis. High RF levels are related to an increased risk of developing RA, which may increase 26 times if they are >100 IU/mL [33], while the presence of the IgA isotype is associated with extra-articular manifestations [34]. Due to RF variable specificity, ACPA testing has been added in the latest RA classification criteria in order to increase the specificity. In RA, 50–70% of patients are positive for ACPAs, which recognize several citrullinated antigens, such as α-enolase, fibrinogen, filaggrin, vimentin, and type II collagen [35,36]. In various diseases, IgG and IgA avidity is associated with different clinical response and, in the case of ACPAs, low avidity IgG is linked to more severe bone damage [37]. About 50% of RA patients have an abnormal serologic profile several years before the development of symptoms. Moreover, elevated serum level of IgM-RF or ACPAs in healthy subjects indicates a high risk for RA onset [38]. Anti-CarP recognize other post-translational modified (PTM)-antigens and often occur together with other antibodies (e.g., ACPAs) with which they can cross-react. Anti-CarP are prognostic of the new RA onset, being detectable years before, and increasing shortly before the RA onset [39], and are predictive of joint destruction, especially in ACPA-negative RA patients [40], and they represent an important serological marker in ACPA-negative RA patients because predictive of more severe disease, as shown by radiological progression [40].

In antibody-positive patients, an increase of IgA plasmablast in peripheral blood is identified, suggesting that mucosal immune responses are involved in early RA pathogenesis [41], as well as the presence of ACPAs and RFs in the subclinical stage [42]. Therefore, Anti-CarPs, ACPAs, and RFs have a predictive role, appearing 1–3 years before RA symptoms onset, and their presence is associated with joint destruction, contributing into RA pathogenesis and development [43]. ACPAs and RFs could enhance the secretion of neutrophil extracellular traps (NETs), which show a pro-inflammatory effect, exposing autoantigens [44]. In RA patients, ACPAs are associated with parenchymal lung abnormalities [45] and may have a role in bone erosion affecting osteoclasts and increasing osteoclast precursor differentiation in vitro and in vivo [46]. This is very important because RA is characterized by chronic inflammation and bone erosion, also caused by immune complexes (ICs) composed of autoantibodies that worsen pro-inflammatory status, leading to complement activation and increased levels of chemokines and cytokines [47,48,49,50]. In a cohort of 200 RA patients, it has been demonstrated that RF testing, followed by ACPA and anti-RA33 analysis, can be a useful workflow in diagnosing the early inflammatory joint damage [51].

De Moel et al. investigated the association between the breadth of the serum autoantibody profile with early and late treatment outcomes in 399 seropositive RA patients enrolled in the IMPROVED (the induction therapy with methotrexate and prednisone in rheumatoid or very early arthritic disease) trial. The seropositive patients with a broader autoantibody profile at baseline exhibited an early treatment response but they did not maintain such favorable outcome over time. The authors suggested that the presence at baseline of multiple autoantibody types, including ACPAs, anti-CARP, RF, and the anti-modified protein antibodies (AMPAs), could improve an early response to DMARDs by stimulating a more active humoral immunity [52].

A study by Martin-Mola et al. suggested that ACPAs, especially when present in high titers, may predict a better response to rituximab and abatacept, but not to anti-TNF agents and tocilizumab [53]. Therapy with these drugs can be complicated by the development of anti-drug antibodies (ADA), since they are proteins and, therefore, inherently immunogenic [54]. The presence of ADA may compromise therapeutic efficacy, leading to a reduction of serum drug levels or even to a neutralization of the drug, causing a loss of clinical response [55,56,57]. ADA development is multifactorial and depends on many factors, even if they are not still clarified. In a study on RA patients receiving infliximab as a second agent, ADA development, low serum infliximab, and RF positivity were more common in females and associated with treatment failure [58]. This finding is interesting, since, as already mentioned, women are more frequently affected by autoantibody-positive autoimmune diseases [59]. In a multicohort analysis evaluating different variables, factors associated with ADA development were longer disease duration, moderate disease activity, and lifetime smoking [60]. Previous studies have shown that 28% of patients treated with adalimumab reported having ADA within 3 years of treatment [56]. ADA have also been observed in up to 44% of patients treated with infliximab in the first 6 months of treatment [61]. On the other hand, in different studies, etanercept has been shown to have mild immunogenicity, reporting an incidence of ADA only in 0–7% of patients [54,62]. A meta-analysis of 17 studies on the immunogenicity of TNF inhibitors reported that ADA were not detected in patients treated with etanercept [63]. Similar results were observed in a recent study in which it was found that all the etanercept-treated patients were negative for ADA, while 31.2% and 17.4% of those treated with adalimumab and infliximab, respectively, were positive for ADA [64]. A meta-analysis of 34 studies enrolling 4273 patients evaluated the clinical response in patients with ADA treated with anti-TNF alpha drugs, showing a significant reduction of response in patients with ADA compared to patients without, especially when treated with infliximab or adalimumab. In this view, early detection of serum ADA levels may improve patients’ management [65]. The current guidelines do not recommend routine monitoring of ADA in cases of primary or secondary failure of anti-TNF therapy. Consequently, when patients show a loss or lack of response to a first anti-TNF alpha, the clinical practice is usually based on switching to a biologic drug with a different mechanism of action, or switching to another anti-TNF alpha, or increasing the dose without changing drug [66]. The decision is often empirical and depends on physician discretion. Several algorithms, which foresee the ADA and therapeutic drug monitoring, have been proposed—they are all based on the hypothesis that immunogenicity assessment may help to guide therapeutic decisions, going beyond the “empirical switching” and obtaining better control of the disease [67]. In a recently proposed algorithm, the measurement of drug levels plays a crucial role. In the first branching of the algorithm, testing ADAs is not recommended because they are not always clinically relevant. In case of treatment failure, they recommend the supplementary ADA test, which may be helpful in personalizing the treatment choice [68].

Table 1 reports the studies, discussed in the text, highlighting the relationship between serum autoantibodies and treatment outcomes in patients with RA.

## 3. Pharmacogenetic Biomarkers

Up until now, there are no data on the role of genetic polymorphism concerning tsDMARDs. On the contrary, several studies highlighted the impact of the pharmacogenetics (PGx) to personalize the therapy with cs- and bDMARDs in patients suffering from RA.

### 3.1. Pharmacogenetics of csDMARDs

Owing to its efficacy, long-term safety, dose-titratable range, and cost-effectiveness, methotrexate (MTX) is still one of the most important drugs used in RA therapy.

MTX is recommend as first-line treatment in patients with RA, both as monotherapy and as an “anchor drug” in association with other DMARDs [7,69]. Following oral administration, it is absorbed from the small intestine via folate transporters and then converted through hepatic and intracellular metabolism into polyglutamated forms (MTX-PGs). Within the cell, MTX-PGs bind to and inhibit dihydrofolate reductase (DHFR) and other folate pathway enzymes required for purine and pyrimidine synthesis [70].

Despite its widespread use, the mechanism by which MTX exerts its therapeutic effects in RA is not fully understood. The reason is that MTX has anti-inflammatory effects, mediated through a variety of molecular pathways that do not necessarily involve the folate antagonism. Multiple mechanisms appear to be involved, including increase and release of adenosine; inhibition of methyl-donor production; generation of radical oxygen species (ROS); downregulation of adhesion molecules levels, eicosanoids, and matrix metalloproteinases; and interference with T cell activity and cytokine secretion [70].

Unfortunately a considerable number of patients with RA (nearly 30–40%) do not respond to MTX adequately, and many of them discontinue the therapy often because of MTX-related adverse drug reactions (ADRs). The most frequently reported ADRs include ulcerative stomatitis, leukopenia, nausea, and abdominal distress but also malaise, undue fatigue, chills and fever, dizziness, and infections [71].

However, at present, there are no biomarkers or models to predict MTX responsiveness so robust as to effectively tailor the MTX treatment in patients with RA.

Many investigations, using various technical approaches, have been proposed with the main aim to stratify the patients on their risk to not respond to MTX. For example, mRNA expression profiling [72,73,74] are considered biomarkers valuable to predict patients’ clinical outcomes before the beginning of the treatment.

In this context, the MTX PGx may be very helpful. Actually, it is well known that, besides disease-specific characteristics, numerous factors, including drug–drug interaction and patient’s genetic background may influence both the MTX efficacy and tolerability [75,76].

Recently, several genetic variants possibly influencing the MTX pharmacokinetics and pharmacodynamics have been explored. The interindividual variability in MTX pharmacokinetics can be partly explained by the presence of polymorphisms (mainly single nucleotide polymorphisms, SNPs) in the genes encoding membrane transporter proteins. MTX is absorbed through active transport by the reduced folate carrier protein 1 (RFC1) and the proton-coupled folate transporter (PCFT), encoded by the human solute carrier family 19 and 46 member 1 (SLC19A1 and SLC46A1), respectively [75,77].

One of these SNPs is *RFC1*-80*G*>A (rs1051266), which results in variable intracellular levels of MTX-PGs, and consequently variable MTX efficacy. It has been shown that patients carrying *RFC1* 80AA genotype responded better to the treatment than *RFC1*-80AG and *RFC1*-80GG individuals, and the probability of remission of RA symptoms was 3.32-fold higher in carriers of *RFC1*-80AA when compared with those with *RFC1*-80GG genotype [78].

As expected, there is a relevant variability depending upon the ethnic population. As matter of a fact, Li et al. found a significant association between *RFC1*-80G>A and MTX efficacy in Asians (*p* = 0.002 for A allele; *p* = 0.003 for AA genotype) but not in Caucasians (*p* = 0.15 for A allele; *p* = 0.05 for AA genotype), and no association with toxicity was found [79]. In contrast, Qiu et al. demonstrated an association between MTX-related toxicity and *RFC1*-80G>A SNP in Caucasian patients (odds ratio (OR) = 1.36, 95% CI = 1.01–1.83, Z = 2.05, *p* = 0.041) [76].

However, the studies included in these metanalyses reported different results also considering study populations belonging to the same ethnicity. Drodzik et al. [78] conducted a prospective study on 174 Caucasian patients affected by RA and treated with a regimen of MTX (7.5–15.0 mg weekly) plus low doses of methylprednisone. The efficacy of treatment was evaluated according to the American College of Rheumatology response criteria (ACR20%) in 12 months, and the safety as number of adverse events. The probability of remission of RA symptoms was 3.32-fold higher in patients with *RFC1*-80AA genotype than in those with -80GG genotype. Adverse events, such as increase of transaminase, were mostly detected in patients with -80AA genotype than in -80GA and -80GG genotypes, but without reaching statistical significance [78]. Conversely, in a retrospective study enrolling 150 Caucasian RA patients, Bohanec-Grabar et al. found an association between the risk of MTX-related toxicity and the presence of *RFC1*-G80A SNP. In fact, patients carrying *RFC1*-80GG genotype had a higher risk for MTX overall toxicity. Gastrointestinal complaints and hepatotoxicity were the most frequent drug adverse events, and such a finding was found also by Lima et al., who demonstrated an increased gastrointestinal MTX-related toxicity associated with the presence of *RFC1*-80G allele in 233 Caucasian RA patients. Differently from Bohanec, these authors found no association between this SNP and overall toxicity [80,81].

Another prospective study performed in Caucasian patients stressed the importance to take into account different variables to evaluate variability response to RA treatment. The research analysed the possible association between several polymorphisms in folate pathway and efficacy of MTX combined with sulfasalazine and hydroxychloroquine in 98 patients with early RA. In line with the results reported by Drodzik et al. [78], the SNP was significantly associated with the status of responder to the therapy. Moreover, patients carrying such a polymorphism combined with *methionine synthase* (*MTR*)-2756A>G or *TYMS*3R-del6 polymorphisms were more likely to have remission of symptoms up to 3 years [82].

Hayashi et al. analyzed 170 Japanese patients with RA. Of the patients, 89 were treated with MTX alone (MTX group), and 81 with MTX plus bDMARDs. The disease activity score (DAS)28 was the same (2.3–2.4) in the two groups. *RFC1*-80A allele frequency was higher in MTX group while more patients carrying the G allele were observed in the MTX plus bDMARDS arm. Not coincidentally, the presence of the G allele may be associated to a poor response to MTX monotherapy that requires the co-administration with a bDMARD [83]. Conversely, another study performed in Japanese RA patients failed to find a significant association between *RFC1*-80A>G, neither with MTX efficacy nor MTX safety [84].

Several others carriers involved in MTX influx have also been examined [77]. One of the most promising pharmacogenes is the *ABC* transporter family members (ATP-binding cassette transporter), coding for the P-glycoprotein (P-gp-1) efflux pump [85,86]. This P-gp, expressed on the membrane of a variety of cells, serves to decrease the intracellular accumulation of various drugs [87]. The SNP *ABCB1* 3435C>T is one of the most investigated polymorphisms because it may influence, especially together with other genetic variants, the response to several drugs, including the antiplatelet clopidogrel [88], statins [89], the antineoplastic agent irinotecan [90], and others.

The SNP *ABCB1* 3435C>T has been related to the clinical course of RA, as well as to the drug response [84,91,92]. However, the available literature data are often contradictory.

Two meta-analyses have investigated the potential of *ABCB1* C3435T in predicting the response to MTX in RA patients. They shared four articles and reached opposite conclusions.

The study by Lee et al, including nine articles involving 1275 RA patients, reported that patients carrying the *ABCB1* C3435T may have an increased risk of MTX-related toxicity (TC vs. TT + CC; OR = 0.483, 95 % CI = 0.259–0.900, *p* = 0.022), but such a SNP does not predict RA susceptibility nor responsiveness to DMARDs [93]. These authors highlighted that a limitation of their study is the small statistic power; therefore, they stressed the need to analyze larger scale studies to validate the association between the *ABCB1* C3435T SNP with efficacy and toxicity in RA. Conversely, the study by He et al., including 12 articles involving more RA patients with respect to the study by Lee et al. (a total of 2014 patients), suggested that the *ABCB1* C3435T might be helpful to predict the response to MTX in Asian patients (additive: OR = 1.64, 95% CI = 1.09–2.48, *p* = 0.019; homozygous model: OR = 2.54, 95% CI = 1.51–4.8, *p* < 0.001; and recessive model: OR = 2.09, 95% CI = 1.37–3.19, *p* = 0.001) but not the MTX-related ADRs [92]. 

Regarding MTX target genes, some polymorphisms in the gene “*TYMS*” encoding TS, which is a key enzyme for DNA synthesis and repair inhibited by MTX-PGs, seem to be related to the response to MTX. The most common genetic variant, located in the enhancer region (TSER), consists of two or three 28-bp tandem repeats (*TYMS*-TSER-2R/3R). It has been reported that individuals carrying -3R allele have higher TYMS mRNA expression than those with -2R and require a higher MTX dosage in order to achieve an adequate therapeutic response [77,94,95].

Different levels of TYMS mRNA has been also considered to understand a possible role of the TSER-2R/3R polymorphism in predicting fluoropyrimidine-related toxicity in oncological patients [96,97]. 

Several meta-analyses focused mostly on SNPs in the gene encoding the 5,10-methylenetetrahydrofolate reductase (*MTHFR*) [76,98,99,100,101], a rate-limiting enzyme in the methyl cycle catalyzing the conversion of 5,10-methylenetetrahydrofolate to 5-methyltetrahydrofolate, which in turn serves as a methyl donor for the methylation of homocysteine to methionine. Variants in the *MTHFR* may interfere with these physiological events, thus leading to unexpected MTX side-effects.

The *MTHFR* C677T (rs1801133) and A1298C (rs1801131) are two SNPs that are well known to affect enzyme activity and drug metabolism. The first results in a thermolabile form of MTHFR with a reduced enzyme activity (30% of wild-type activity for TT carriers) and hyperhomocysteinemia, and increased toxicity of MTX has been associated with such a polymorphism [95].

Moreover, the *MTHFR* A1298C leads to lower enzyme activity and hyperhomocysteinemia but without resulting in a thermolabile protein and, in contrast to the *MTHFR* 677C>T, it does not seem to increase the frequency and severity of MTX-associated toxicity [95].

Additionally in this case, the available data are controversial. For example Bohanec-Grabar et al. observed that carriers of *MTHFR* A1298C SNP showed a decreased overall MTX toxicity but no effects on MTX efficacy [87].

Moreover, the already mentioned HLA-DRB1-shared epitope is linked to the response to MTX. In particular, the presence of HLA-DRB1*04 allele in combination with anti–cyclic citrullinated peptide (anti-CCP) antibodies has been associated with development of MTX resistance and it may justify the use of an anti-TNFα agent in patients with early RA [25,102]

Other genetic variants in genes involved in MTX transport and metabolism (e.g., *FOLR1, FPGS* and *GGH*), as well as in purine synthesis (e.g., *ATIC, AMPD1, ADA,* and *ADORA2A*) or adenosine signalling (*AMPD1, ATIC, ITPA, MTR,* and *MTRR*), have been explored, often with contrasting results [86,94].

For example, Wessels et al. found that patients carrying *AMPD1* 34T allele, or *ATIC* 347CC or *ITPA* 94CC genotypes were more likely to have a good clinical response [103], while Weisman et al. reported that *ATIC* 347GG genotype was associated with an increased risk of MTX-related gastrointestinal ADRs [104]. In a recent meta-analysis, Lee et al. investigated the potential of the *ATIC* 347C/G polymorphism in predicting efficacy and safety of MTX. Examining nine comparative studies including 1056 RA patients, the authors found that *ATIC* 347 GG + GC genotype was associated with the lack of response to MTX (OR = 1.884, 95% CI = 1.236–2.873, *p* = 0.003) and with MTX-related toxicity in Caucasian RA patients (OR = 1.741, 95% CI = 1.080–2.806, *p* = 0.023) [105].

To date, only two genome-wide association studies (GWASs) have been carried out to investigate genetic predictors of response to MTX IN RA patients [106,107]. Taylor et al combined data from two consortia to carry out a GWAS on the response to MTX in 1424 early RA patients. The authors found that no SNP reached significance for any outcome measure [106]. Senapati et al. performed a GWAS in 457 RA patients on MTX monotherapy, classified as good responders (GRs, *n* = 297) and poor-responders (PRs, *n* = 160) according to DAS28 values. None of the association signals reached genome-wide significance (*p* < 5 × 10^−8^). However, seven novel risk variants with a suggestive association (*p*-value between 5 × 10^−8^ and 5 × 10^−5^) were identified. These included three genes, namely, ADP-ribosylation factor-like 14; protein phosphatase, Mg^2+^/Mn^2+^ dependent, 1L (ARL14|PPM1L); protein tyrosine phosphatase, receptor type M (PTPRM); and bone morphogenetic protein 2 (BMP2) and four intergenic regions. All the markers are common among RA patients and confer risk for poor response to MTX. Only eight SNPs from five candidate genes (MTRR, DHFR, GGH, FPGS, and TYMS) were found to be associated with at *p* ≤ 0.05, of which only five SNPs from DHFR, FPGS, and TYMS withstood gene-wide testing. The most significant allelic and genotypic association was present in the intron-2 of TYMS (rs2244500-G>A; *p* allelic = 0.005, OR = 1.48; *p* genotypic = 0.004, OR = 1.48). Moreover, of the 334 SNPs included by using a support vector machine (SVM) approach to identify potential interactions, three novel significant genes, namely, adenosine deaminase RNA-specific B2 (ADARB2), WW domain-containing oxidoreductase (WWOX), and neuroligin 1 (NLGN1) for MTX (poor) response were found. An additional four suggestive risk genes, namely, ROBO2, GALR1, DNAH8, and BMP2 were also identified. The authors observed that at all the SVM- and GWAS-identified genes showed genetic interactions and were co-expressed with the three well-known candidate genes DHFR, FPGS, and TYMS and also notably involved in a common network. At the TYMS locus in addition to the top index SNP(rs2244500) observed in the candidate GWAS, three stronger association signals at rs1824993, rs71363250, and rs11662367 that localized in intron-2 of COLEC12, intron-9 of ENOSF1, and 10 kb downstream of ADCYAP1, respectively, were found. Notably, all the markers at this locus that are in high linkage disequilibrium (LD) with the index SNP were present only within a B48-kb (659.2–707.5 Mb) region, falling within the TYMS-ENOSF1 gene region defined by a strong LD block. Therefore, the authors suggested that the significant association of variants in only three genes (i.e., DHFR, FPGS, and TYMS) and the marginal association of GGH and MTRR may be interpreted to reiterate the contribution of these genes from the pharmacokinetic pathway toward MTX (poor) response in RA [107].

Despite considerably large analyzed cohorts, these studies failed to identify genetic markers usable in clinical practice. However, the lack of univocal consensus from pharmacogenetic studies is unsurprising, given the pleiotropic nature of the RA and the heterogeneity of the enrolled study populations. It is necessary to perform large-scale prospective studies, which should be more homogenous, in order to clarify the impact of pharmacogenetics in determining the MTX response in RA.

### 3.2. Pharmacogenetics of Other csDMARDS

Leflunomide (LEF) is used in patients with a contraindication to MTX. It is a prodrug that is converted into the active form A77 126 (teriflunomide). This form inhibits the dihydroorotate dehydrogenase (DHODH), an enzyme involved in the biosynthesis of pyrimidines. More than 50% of patients fail to respond to this treatment because of genetic polymorphisms.

It has been reported that the efficacy of the therapy with LEF is superior in men in comparison with women. In fact, estrogens may contrast the anti-inflammatory action of leflunomide by enhancing cytokines production.

Dziedziejko et al. [108] investigated the association between polymorphisms in the genes coding for two estrogen receptors (i.e., *ESR1* and *ESR2*) and leflunomide treatment outcome in female RA patients. The authors found that the patients who were homozygous AA for *ESR1*-351A>G (rs9340799) and homozygous TT for *ESR1*-397T>C (rs2234693) responded to the therapy. The subjects bearing ESR1 rs9340799-rs2234693 A-T haplotype showed a better response, while those with ESR1 rs9340799-rs2234693 G-C haplotype demonstrated a worse response. There was no statistically significant association with response to treatment considering *ESR2* rs4986938 and rs1256049 polymorphisms [108]. Similarly, Cutolo et al. [109] demonstrated that sex hormones interfere with expression/production of pro-inflammatory cytokines by activated macrophages (including RA synovial macro-phages) and modulate the downregulatory activity exerted by drugs such as leflunomide on the immune/inflammatory reaction. The authors found that 17b-estradiol and testosterone respectively seem to contrast and to synergize the LEF downregulatory activity [110]. In another study, Dziedziejko et al. [111] examined the association between CAG repeat polymorphism in the androgen receptor (AR) gene and response to therapy in women with RA treated with LEF. The AR gene, present on the X chromosome, contains a highly polymorphic CAG (glutamine) repeat in the region encoding NH2-terminal transactivation domain, which normally varies between 9 and 37 repeats (approximately 10–30 in Caucasians) [112,113]. CAG length ((CAG)n) is inversely associated with the AR transcriptional activity, such that, having longer (CAG)n, it reduces AR transcriptional activity and subsequent intracellular androgenic activity [110,113].

The authors studied 114 women diagnosed with RA and treated with LEF 20 mg daily, with a 12-month follow-up. No statistically significant associations between CAG repeat polymorphism in the AR gene and improvement of disease activity parameters was found, suggesting no correlation between CAG repeat polymorphism in the AR gene and response to treatment with LEF in women with RA [111].

Pawlik et al. reported the relationship between *DHODH* 19C>A SNP and the efficacy of LEF treatment in 147 RA patients. Clinical improvements, evaluated according to the American College of Rheumatology 20% and 50% response criteria (ACR20 and ACR50), were found in C allele carriers as opposed to in those carrying the allele A [114].

Some genetic variants possibly related to response to LEF in RA patients have been found in the genes coding for CYP1A2, CYP2C19, and CYP2C9 isoenzymes that are all involved in the bioactivation of the drug. It seems that the CC genotype of *CYP1A2*-164A>C SNP is significantly associated to an increased risk of developing LEF toxicity, mainly gastrointestinal and neutropenia [115,116].

Besides LEF, sulfasalazine represents another therapeutic option in patients who are non-responders to MTX. It is also a prodrug converted in its active form by coliform bacterial enzyme, azoreductase, to sulfapyridine and 5-aminosalicylic acid (5-ASA). The mechanism of action is not clear, but it seems that sulfasalazine and its metabolites interfere with folate and purine metabolism by inhibiting DHFR and thiopurine methyltransferase. Polymorphisms in genes coding for N-acetyl transferase 2 (*NAT2*) and *ABCG2*, involved in drug metabolism and elimination, respectively, could influence both response to sulfasalazine and its related toxicity. Wiese at al. analyzed these polymorphisms in RA patients treated with a DMARD regimen including sulfasalazine. A total of 26 out of 140 patients carrying loss of function allele A of the *ABCG2* 421C>A SNP showed a beneficial effect after 12 months of treatment (OR = 3.34, 95% CI = 1.18–9.50, *p* = 0.024). Instead, the toxicity profile appeared to be linked to the *NAT2* genotype (hazard ratio = 1.74, 95% CI = 1.01–3.21, *p* = 0.044). In fact, carriers of loss of function (LoF) allele of *NAT2* 341T>C and *NAT2* 282C>T, thus classified as intermediate or slow acetylators, developed more toxicities than rapid acetylators within 12 months of treatment [117].

**Figure 1:**
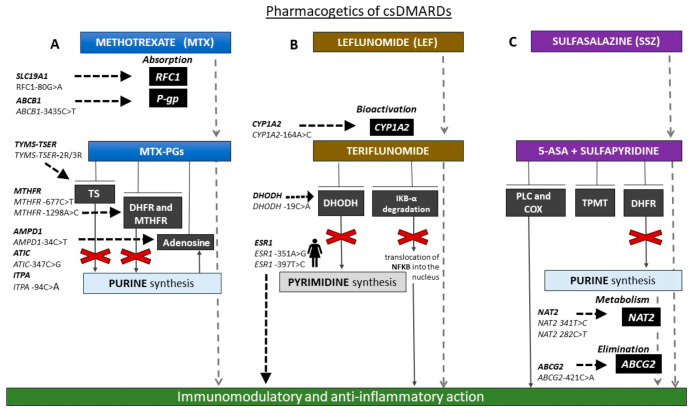
Schematic representation of the main pharmacogenetic biomarkers associated with conventional synthetic disease-modifying antirheumatic drug (csDMARD) treatment. (**A**) Methetrexate (MTX) is absorbed by reduced folate carrier 1 (RFC1) transporter, encoded by human solute carrier family 19 member 1 (SLC19A1). Patients with RFC1-80AA genotype responded better to MTX than -80AG and -80GG individuals. ATP-binding cassette subfamily B member 1 (ABCB1)-3435CT single nucleotide polymorphism (SNP) may influence the MTX absorption and therapy response. MTX is converted into MTX-polyglutamates (MTX-PGs), which in turn inhibit folate pathway enzymes thymidilate synthase (TS), dihydrofolate reductase (DHFR), and methylene tetrahydrofolate reductase (MTHFR) and increase adenosine release. Individuals carrying -3R allele of the genetic variant in the enhancer region of thymidylate synthase (TYMS; thymidylate synthase enhancer region (TSER)), encoding TS (TYMS-TSER-2R/3R) have higher TYMS mRNA expression than those with -2R and require a higher MTX dosage. MTHFR -677CT and -A1298AC SNPs lead to lower MTHFR enzyme activity. Moreover, MTX response and toxicity may be affected by SNPs in genes involved in adenosine signaling (adenosine monophosphate deaminase 1 (AMPD1)-34C>T, 5-aminoimidazole-4-carboxamide ribonucleotide formyltransferase/inosine monophosphate cyclohydrolase (ATIC)-347C>G, inosine triphosphate pyrophosphatase (ITPA)-94C>A). (**B**) Leflunomide (LEF) is converted into the active form, teriflunomide, mainly by CYP1A2. CC genotype of CYP1A2-164AC SNP is associated with increased LEF toxicity. Teriflunomide inhibits dihydroorotate dehydrogenase (DHODH; enzyme involved in the biosynthesis of pyrimidines) and nuclear factor of kappa light polypeptide gene enhancer in B cells inhibitor alpha (IκBα) degradation. DHODH-19CC patients respond better than -19AA. The efficacy of LEF is superior in patients with ESR1 rs9340799 AA and ESR1 rs2234693 TT genotypes. (**C**) Polymorphisms in genes coding for N-acetyltransferase 2 (NAT2) and ATP-binding cassette subfamily G member 2 (ABCG2), involved, respectively, in drug metabolism and elimination, could influence response and safety of sulfasalazine. Patients carrying loss of function (LoF) allele A of the ABCG2 -421CA SNP respond better than carriers of LoF allele of NAT2-341TC, and -282CT is associated with high-risk toxicity. csDMARDs, conventional synthetic disease-modifying antirheumatic drugs; MTX, methetrexate; SLC19A1, human solute carrier family 19 member 1; RFC1, reduced folate carrier 1; P-gp, P-glycoprotein; ABCB1, ATP-binding cassette subfamily B member 1; MTX-PGs, MTX-polyglutamate: TYMS-TSER, thymidylate synthase-thymidylate synthase enhancer region; TS, thymidilate synthase; DHFR, dihydrofolate reductase; MTHFR, methylene tetrahydrofolate reductase; AMPD1, adenosine monophosphate deaminase 1; ATIC, 5-aminoimidazole-4-carboxamide ribonucleotide formyltransferase/inosine monophosphate cyclohydrolase; ITPA, inosine triphosphate pyrophosphatase; DHODH, dihydroorotate dehydrogenase; IκBα, nuclear factor of kappa light polypeptide gene enhancer in B cells inhibitor, alpha; NFκB, nuclear factor kappa-light-chain-enhancer of activated B cells; ESR1, estrogen receptor 1; TPMT, thiopurine methyltransferase; 5-ASA, 5-aminosalicylic acid; TPMT, thiopurine S-methyltransferase; NAT2, N-acetyltransferase 2; ABCG2, ATP-binding cassette subfamily G member 2.

Figure 1 reports the main pharmacogenetic biomarkers associated with csDMARD treatment.

### 3.3. Pharmacogenetics of bDMARDs

Biological DMARDs are now frequently administered in patients with RA both as alternative treatment and together with csDMARDs. However, accumulating evidence demonstrates therapeutic response variability also for this class of drugs. Therefore, given the variety of bDMARDs now available and their high costs, the identification of pre-therapeutic predictors of response is becoming a priority.

It has been reported that 25–30% of patients with RA do not respond adequately to TNFα inhibitors [118].

The anti-TNFα PGx is not yet used in daily clinical practice. Indeed, the results are promising but yet inconclusive.

Several studies have shown that the presence of polymorphisms in the genes coding for *TNFα* and *TNF* receptor (TNFR) could influence the response to treatment. Among them, three studies evaluated the association between *TNFα* 308A>G (rs1800629) SNP and clinical response to infliximab [119], etanercept [120], and adalimumab [121], independently. All of these studies showed that RA patients with a *TNFα*-308 G/G genotype responded to biological drugs better than patients with -308 A/G or -A/A genotypes, suggesting that *TNFα*-308A>G genotyping may be a useful tool for predicting the response to anti-TNF agents. Similar results were obtained in the meta-analysis [122] by Zeng et al. that, including 15 studies with a total of 2127 RA patients, suggests that *TNFα*-308G allele plays a major role in guiding the response to anti-TNFα treatment (OR = 1.87, 95 % CI = 1.26–2.79) [122].

Maxwell et al. [123], in a cohort of RA patients treated with anti-TNF drugs (455 with etanercept and 450 with infliximab), showed that *TNFα*-308AA genotype was associated with a significantly poorer response compared with *TNFα*-308GG among the patients treated with etanercept but not in those with infliximab [123]. In addition, Kang et al. proposed another polymorphism in the *TNFα* gene, (i.e., *TNFα*-857C/T SNP, rs1799724) as a biomarker of response to etanercept. These authors analyzed 70 RA patients and found that those carrying *TNFα*-857T allele responded better than the homozygous for *TNFα-* 857CC [124].

Miceli-Richard et al. [125] stressed the importance to concomitantly consider more than one genetic variation. These authors performed a large pharmacogenetic study including 388 Caucasian RA patients, providing evidence that patients who were homozygous for a single *TNF* locus haplotype (-238G/-308G/-857C) showed a lower response to adalimumab, mainly when such a drug was combined with MTX [125].

Unlike all previously mentioned studies, Ongaro et al. [126] did not find a significant association between TNFα-308G>A polymorphism and clinical response. On the contrary, these authors report that another SNP, the TNF receptor II (TNFRII) 676TG, could be associated to anti-TNF outcomes. 

In particular, they analyzed a total of 105 RA patients (55 treated with etanercept, 40 with infliximab, and 10 with adalimumab), finding that the TNFRII-676TG genotype was significantly associated with lower ACR response compared with -676TT genotype, 3 and 12 months after the beginning of the therapy [126].

Apart from the already mentioned study by Ongaro et al. [126], other researchers investigated possible association between polymorphisms within the *TNF* receptors (*TNFR*) genes and responsiveness to TNFα blockers. Among them, Swierkot et al. [127] analyzed five SNPs in the *TNFα* and TNFR genes (*TNFα*-G308A, -G238A, and -C857T; *TNFR1A*-G36A and *TNFR1B*-T676G), finding that, after a 6-month therapy, RA patients with *TNFR1A*-36AA genotype showed a better response than those who were *TNFR1A*-36GG homozygotes. In addition, according to the results of Kang et al. [124], patients bearing *TNFα*-857TT genotype were better responders to anti-TNF treatment than the *TNFα*-857CC homozygotes [127]. In a multicenter study, the association between SNPs in the gene encoding TNFR superfamily member 1B (*TNFRSF1B*) and treatment response was investigated in 596 anti-TNF-naïve RA patients [128]. The results showed that subjects who were carriers of the *TNFRSF1B*-676GG genotype had an increased risk of having a worse response to TNF blockers. However, this association only reached a marginal statistical significance and was not confirmed through a meta-analysis [128].

A meta-analysis investigating the impact of the *TNFRSF1B* polymorphisms in influencing the therapy with anti-TNF was conducted by Chen et al. [129]. These authors investigated the association between *TNFRS1B*-T676G and *TNFRSF1A*-A36G SNPs with responsiveness to anti-TNF therapy in autoimmune diseases (RA, psoriasis, and Crohn’s disease). An association between the TNFRSF1B (rs1061622) allele and non-responders to TNF inhibitors in RA was found (T/G OR = 0.69, 95% CI = 0.48–0.99, *p* < 0.05). The patients who were carriers of *TNFRSF1B*-T allele were the better responders than the others. Notably, these results are consistent with those obtained by Ongaro et al. [126], while they are in contrast with those reported by Swierkot et al. [127].

Besides polymorphisms in *TNF* and *TNFR* genes, the response to anti-TNF agents can be modulated by other genetic variants.

Cui et al. analyzed a GWAS meta-analysis of nearly 2 million variants in 2706 patients suffering from RA and treated with etanercept, infliximab, or adalimumab. Primary endpoints were the change in disease activity score based on DAS28 evaluated up to 3–12 months from the beginning of the therapy [130]. When the authors tried to analyze all patients without making distinctions about the drug used, no significant association with therapy response was provided. Then, when they separated the data on the specific anti-TNF used, considering their different pharmacodynamics and biochemical characteristics, a locus on chromosome 1q23 achieved a very high statistical significance for the patients treated with etanercept but not for those with infliximab or adalimumab. The top SNP was *CD84* rs6427528 (*p* = 8 × 10^−8^) in etanercept, but not in the infliximab or adalimumab subsets (*p* > 0.05), able to influence the immune related gene *CD84* by disrupting a transcription factor site motif in its 3′-untranslated region (UTR). Interestingly the expression protein levels of CD84 correlated positively with disease activity (assessed by DAS28) in patients (only those of European ancestry) treated with etanercept. The results of this study highlight the importance to investigate the response to specific drug treatment and are consistent with the fact that RA patients who did not adequately respond to one TNF inhibitor may still benefit from another [130]. Plant et al. analyzed the GWAS data conducted from Wellcome Trust Case Control Consortium in 566 anti-TNF-treated RA patients [131]. They found that the SNP (rs173011249) was significantly associated with improved response (coefficient −0.27, *p* = 5.67 × 10^−5^). This involved an intronic variant located in the gene *EYA4* (eyes absent homolog 4) that may induce the expression of interferon β (IFN-β) whose high levels have been correlated with a poor clinical response to infliximab [132]. Another gene candidate to predict the response to anti-TNF is *FCGR2A*, which codes for immunoglobulin gamma Fc region receptor II-a, principally expressed in dendritic cells and macrophages. TNFα inhibitors possess an IgG1 Fc portion with binding affinity to FCGR; therefore, it is unsurprising that variation in Fc regions could influence the response to the therapy [133]. *FCGR2A* rs1801274 SNP has been associated with variable response to anti-TNF therapy in RA patients [134]. Recent studies suggest that *FCGR2A* genetic variations could predict the response to the therapy with TNF inhibitors, but it depends on the type of the agents administered. In fact, more convincing results involved patients treated with infliximab [134,135], while no association with the response to etanercept was found [135,136]. Avila-Pedretti et al., in a cohort of 348 RA patients, observed a statistically significant association between *FCGR2A* and the clinical response to adalimumab. Moreover, *FCGR2A* was significantly associated with the response to infliximab but only in patients positive for anti-CCP, and the lack of association between *FCGR2A* and etanercept was confirmed [137]. Bek et al. performed a systematic review with meta-analysis to evaluate the status of the anti-TNF PGx. considering 47 studies, they evaluated 19 polymorphisms from candidate gene and GWA studies, and other 6 genetic variants from their meta-analysis. In total, 25 single nucleotide polymorphisms were found to be associated with clinical response to anti-TNF treatment. Such genetic variants are located in genes involved in T cell function, nuclear factor kappa-light-chain-enhancer of activated B cells (NFκB), and TNF signaling pathways, including *CTCN5, TEC, PTPRC, FCGR2A, NFKBIB, IRAK3,* and *FCGR2A* [138].

*FCGR2A* is considered a pharmacogene also in the context of treatment with other bDMARDs such as tocilizumab and rituximab. Morales et al. investigated the role of polymorphisms in genes encoding the surface receptors *FCGR2A* and *FCGR3A* in influencing the response to tocilizumab and rituximab in 142 patients with RA previously treated with csDMARDs. These genetic variants altered the affinity of FCRs to constant region (Fc) of the bDMARDS. Patients bearing the lower-affinity genotype (i.e., *FCGR3A* rs396991-TT), probably lowering plasmatic clearance of tocilizumab, demonstrated a higher response to this drug. Conversely the best response to rituximab was found in patients carrying *FCGR3A* rs396991-G allele, associated with a high affinity of FCR for the Fc region, maybe by increasing cellular immunological response [139]. Moreover, besides *FCGR3A* rs396991 polymorphism, the response to both bDMARDs tested in this study depended also on higher baseline DAS28, a smaller number of previous treatments with biologic drugs, and the presence of serum anti-CCP and RF value, according to previous literature data [139,140].

**Figure 2:**
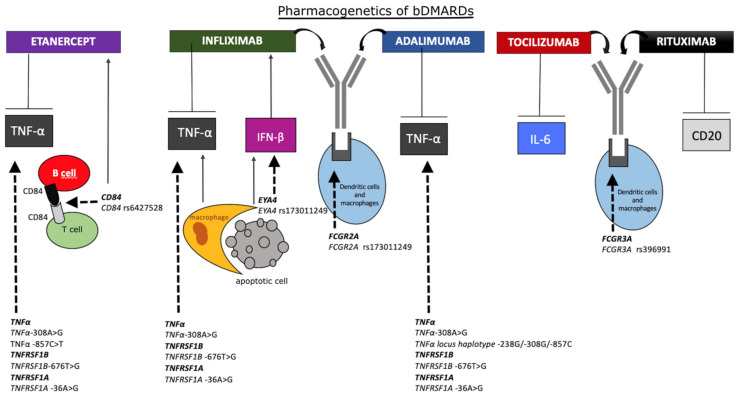
Schematic representation of the main pharmacogenetic biomarkers associated with bDMARDs treatment. Rheumatoid arthritis (RA) patients with tumor necrosis factor α (TNFα)-308 G/G genotype may respond to anti-TNFα drugs better than patients with -308 A/G or -A/A genotypes. TNFα -857C>T and CD84 rs6427528 SNPs also affect the treatment with etanercept. TNFα-308AA genotype is associated with a poorer response compared with TNFα-308GG, and CD84 rs6427528 SNP is positively correlated with disease activity in patients treated with etanercept. In apoptosis, DNA from dead cells is digested by DNase II in the macrophages after they are engulfed. Interferon β (IFN-β) and TNFα are produced from the macrophages carrying undigested DNA. Eyes absent homolog 4 (EYA4), originally identified as a co-transcription factor, stimulates the expression of IFN-β in response to undigested DNA of apoptotic cells, worsening the response to infliximab. The SNP rs173011249 in EYA4 gene is associated with improved therapy response. Treatment with infliximab and adalimumab may be affected by fragment crystallizable region of immunoglobulin (Ig)G receptor 2A (FCGR2A) rs173011249 in gene coding for immunoglobulin gamma Fc region receptor II-a. Patients bearing FCGR3A rs396991-TT genotype, probably lowering plasmatic clearance, respond better to tocilizumab. Rituximab is more efficacious in patients carrying FCGR3A rs396991-G allele, associated with higher affinity of FCR for the Fc region. bDMARDs, biologics disease-modifying antirheumatic drugs; TNFα, tumor necrosis factor α; TNFRSF-1B/1A, TNF receptor superfamily member 1B/1A; IFN-β, interferon β; EYA4, eyes absent homolog 4; FCGR-2A/3A, fragment crystallizable region of IgG receptor 2A/3A; IL-6, interleukin-6.

Figure 2 reports the main pharmacogenetic biomarkers associated with bDMARDs treatment.

## 4. Conclusions

RA is a very complex disease characterized by numerous interactions between genetic and environmental factors.

In recent years, several biomarkers have been proposed to personalize the therapy. Unfortunately, a magic bullet does not exist, as several patients’ characteristics (such as gender, drug pharmacokinetics, previous pharmacological treatments, comorbidity, and polypharmacy) are deeply interconnected. For instance, RF, ACPAs, and ADA autoantibodies could also have a role in guiding the choice of treatment. Although the current guidelines do not recommend their routine monitoring, accumulating evidence confirms their predictive potential.

Pharmacogenetic testing may be of great help to identify patients at high risk of treatment failure and to choose the treatment predicted to have the highest probability of success. However, DMARD pharmacogenetics has not yet been implemented in daily clinical practice because no single polymorphism has reached a satisfactory level of evidence. The genetic variants explored until now, although promising predictive factors, do not fully explain susceptibility to the disease nor whether and how a patient will respond to a specific treatment. Again, the individual’s susceptibility to treatment depends on many factors. Therefore, the influence of a single gene is usually very limited. That makes it necessary to concomitantly evaluate the presence of various polymorphisms and haplotypes and, more generally, to set up complementary strategies taking into account all the measurable variables that act around the congenital background.

In this context, models integrating demographic, clinical, biochemical, and genetic data are needed to enhance the predictive capacity of specific factors singularly considered.

Such predictive models will allow a personalized approach to both pharmacological and nonpharmacological therapy currently used in patients with RA and other chronic diseases in light of multidisciplinary patient management.

## Figures and Tables

**Table 1:** Studies on association between autoantibodies and treatment outcomes in patients with rheumatoid arthritis.

References	Pts (*n*)	Biomarkers	Conclusions of the Study
Lard et al. (2002) [27]	361	HLA class II antigens	An early and aggressive treatment with DMARDs, such as MTX, sulfasalazine, and chloroquine can regulate the immune process, maybe by preventing a secondary release of autoantigens, by modulating the autoantigen presentation by APCs to the CD4+ T cells, and/or by inhibiting the response of T cells. However, this approach is mainly effective in a subgroup of patients with *shared epitope* positivity.
de Moel et al. (2018) [52]	399	IgG, IgM, and IgA isotypes for ACPA and anti-CarP, IgM, and IgA RFs; AMPAs	The presence at baseline of multiple autoantibody types could improve the early response to drug therapy, reflecting a more active humoral immunity. The impact of the baseline autoantibody profile on treatment outcomes diminishes over time.
Pascual-Salcedo et al. (2011) [57]	85	ADA	The presence of ADA, like anti-infliximab Abs, may compromise efficacy and safety of the therapy. The formation of anti-infliximab Abs is associated with appearance of infusion reactions, discontinuation of therapy, and poor clinical response.
Hambardzumyan et al. (2019) [58]	128	ADA, RF	ADA development, low serum infliximab, and RF positivity were more common in females and were associated with treatment failure.
Quistrebert et al. (2019) [60]	366	ADA	In RA patients treated with adalimumab or infliximab and co-treated with MTX, longer disease duration, moderate disease activity, and lifetime smoking were all factors associated with ADA development.
Bartelds et al. (2011) [56]	272	ADA	The production of ADA is associated with a negative outcome. Patients with ADA against adalimumab discontinue therapy earlier and more frequently and less often achieved remission. The presence of ADA influenced serum adalimumab concentrations.
Bendtzen et al. (2006) [61]	106	ADA	Up to 44% of patients treated with infliximab developed ADA in the first 6 months of treatment. ADA development is associated with increased risk of infusion reaction and treatment failure.
Dore et al. (2007) [62]	222	ADA	ADA, all non-neutralizing Abs, were detected in few patients and their presence did not affect etanercept safety or efficacy, except for few cases of serious adverse events (6.3%) and serious infections (2.3%).
Garcês et al. (2012) [63]	1801	ADA	In patients treated with anti-TNFα, the development of ADA reduces the drug efficacy. This event can be avoided by administration of other immunosuppressive agents.
Moots et al. (2017) [64]	595	ADA	All patients treated with etanercept did not develop ADA, while ADA were present in patients treated with adalimumab and infliximab. Patients negative for ADA generally showed better clinical outcomes than those who were ADA-positive.
Pecoraro et al. (2017) [65]	4273	ADA	ADA reduced drug response in patients treated with anti-TNFα, especially infliximab or adalimumab.

DMARDs: disease-modifying antirheumatic drugs; ACPAs: anti-citrullinated protein antibodies; anti-CarP: anti-carbamylated protein antibodies; RF: rheumatoid factor; AMPAs: acetylated peptides (anti-modified protein antibodies); ADA: anti-drug antibodies; RA: rheumatoid arthritis; MTX: methotrexate.
